# Exploring Different Virtual Screening Strategies for Acetylcholinesterase Inhibitors

**DOI:** 10.1155/2013/236850

**Published:** 2013-11-04

**Authors:** Nibha Mishra, Arijit Basu

**Affiliations:** Department of Pharmaceutical Sciences, Birla Institute of Technology, Mesra, Ranchi 835215, India

## Abstract

The virtual screening problems associated with acetylcholinesterase (AChE) inhibitors were explored using multiple shape, and structure-based modeling strategies. The employed strategies include molecular docking, similarity search, and pharmacophore modeling. A subset from directory of useful decoys (DUD) related to AChE inhibitors was considered, which consists of 105 known inhibitors and 3732 decoys. Statistical quality of the models was evaluated by enrichment factor (EF) metrics and receiver operating curve (ROC) analysis. The results revealed that electrostatic similarity search protocol using EON (ET_combo) outperformed all other protocols with outstanding enrichment of >95% in top 1% and 2% of the dataset with an AUC of 0.958. Satisfactory performance was also observed for shape-based similarity search protocol using ROCS and PHASE. In contrast, the molecular docking protocol performed poorly with enrichment factors <30% in all cases. The shape- and electrostatic-based similarity search protocol emerged as a plausible solution for virtual screening of AChE inhibitors.

## 1. Introduction

Acetylcholinesterase (AChE; EC 3.1.1.7) terminates signaling at cholinergic synapses by rapid hydrolysis of the neurotransmitter acetylcholine [1]. It is a validated target for the treatment of the Alzheimer's disease (AD). It is the only target that has provided the few palliative drugs presently marketed for the treatment of the AD [2]. AChE inhibitors are also used for the treatment of Glaucoma [3], Myasthenia gravis [4], and so forth. 

AChE inhibitors are chemically diverse; the active site of AChE is multifaceted and complex in architecture allowing numerous structurally diverse ligands to bind to different subsites [5], thereby, limiting the application of structure based approaches for a universal virtual screening solution. Though many groups [6–8] have reported the application of structure based approaches to AChE, all the studies are focused on exploring a specific set of analogs rather than finding a universal solution.

In this study, we have explored both ligand-based and structure based approaches for virtual screening of AChE. Ligand based approaches such as similarity search and pharmacophore mapping were used whereas molecular docking was used as a structure based approach. The following virtual screening tools were used for this study: (a) molecular docking using AutoDock and Glide [9], (b) similarity search using ROCS [10] and EON [11], and (c) PHASE-Shape based module and PHASE-pharmacophore search module. 

## 2. Material and Methods

### 2.1. Dataset Preparation and Query Selection

Known ligands and decoys set for AChE as reported in the directory of useful decoys (DUD) [12] was used. The latest structural databases were downloaded directly from http://www.dud.docking.org/ (DUD release 2, October 22, 2006) in mol2 file format. The DUD dataset is a well-defined and unbiased dataset of annotated active compounds and decoys for the validation of virtual screening. Multiconformers for the dataset were then created using OMEGA [13]. The ligand structures used as queries were extracted from experimentally cocrystallized structures obtained from the http://www.rcsb.org/ PDB IDs: 1ax9 (edrophonium), 1eve (donepezil), 1gpk (huperzine), 1gqr (rivastigmine), and 1odc (tacrine).

### 2.2. Structure Based Docking

#### 2.2.1. Protein Preparation

 PDB code 1b41 was downloaded (http://www.rcsb.org/) and visually analyzed. All the protein structures were initially corrected using MolProbity [14] interactive server. The resulting structure was then further refined using Schrödinger protein preparation wizard. The ionizable residues were set to their normal ionization states at pH 7, and a restrained energy minimization (relatively higher convergence threshold of a gradient to <0.3 kJ/Å-mol) was performed using OPLS2005 force field.

#### 2.2.2. Ligand Preparation

For docking studies all the ligands were energy minimized in the Macromodel minimization panel using the OPLS-2005 force field and GB/SA water model with a constant dielectric of 1.0. Polak-Ribiere first derivative, conjugate gradient minimization was employed with maximum iterations of 1000 and convergence threshold of a gradient to <0.05 kJ/Å-mol. LigPrep2.0 module of Schrödinger was used to generate possible ionization states at target pH 7.0 ± 2.0. All possible tautomeric states at this pH were also generated using the tautomerizer module of LigPrep2.0. The resulting structures were saved in ∗.mae format for docking using Glide and ∗.pdb format for docking using AutoDock. 

#### 2.2.3. Docking

All the docking experiments were performed with AutoDock4.0 and Glide. A grid size of 110 × 110 × 110 centered on the ligand was used. For Auto Dock, Lamarckian Genetic Algorithm was employed as the docking algorithm. For making the virtual screening protocol automated a separate script was written and validated [15]. The docking parameters used are as follows: number of genetic algorithm (GA) runs: 10, population size: 150, maximum number of evaluation: 2500000, and maximum number of generation: 27000. Glide standard precision mode was used for the current docking study. 5000 poses were used for passing through initial Glide screening. Scoring window for keeping initial poses was kept at 100 poses. Best 400 poses were chosen for energy minimization during docking; a distance dependent dielectric constant of 2.0 and maximum number of energy minimization steps of 100 were used. All the docked poses were then clustered based on heavy atom RMSD clustering, with a maximum cutoff of 2.0 Å. 

### 2.3. Similarity Search

ROCS shape-based virtual screening: Multiconformer files, which were generated by OMEGA, were saved in oeb.gz format. These generated multi-conformational files were used as input database for performing Rapid Overlay of Chemical Structures (ROCS) [10] similarity search. ROC is designed to carry out large-scale 3D database searches. It performs similarity searches by using a shape-based superposition method that finds the similar but nonintuitive compounds. It uses only the heavy atoms of a ligand ignoring the hydrogens. The output files of the similarity search were then ranked according to their combo score (chemistry and shape search score). 

EON electrostatic similarity-based virtual screening: ROCS output structures (oeb.gz) were used as input for analysis using EON. EON [11] calculates the Electrostatic Tanimoto between each database molecule and the query (ROCS overlay hits). EON does not perform any overlay or alter the input orientation of the structures. It calculates new partial charges for the input structures using MMFF94. The output files were clustered according to the EON (ET_combo) score.

PHASE-Shape based similarity: similar to the ROCS program PHASE-Shape program [16, 17] was used to screen a database, based on the shape of any query. The shape search algorithm in PHASE can treat all atoms as equivalent, or it can incorporate information on atom types as part of the search. Searching on atom types favors alignments that superimpose atoms of the same type. In the current study, Macromodel atom types were assigned to all the queries and volume scoring was used to cluster the molecules.

### 2.4. Pharmacophore Mapping

PHASE version 3.0 was used for pharmacophore elucidation. For this dataset, we performed the PHASE procedure with six built-in types of pharmacophoric features: hydrogen bond acceptor (A), hydrogen bond donor (D), hydrophobe (H), negative ionizable (N), positive ionizable (P), and aromatic ring (R). The graphical user interface of maestro was used. Ligands were processed with the LigPrep program to assign protonation states appropriate for pH 7.0. 

Conformer generation was carried out with the Macromodel. Potentials were computed using the OPLS2005 force field. The default pharmacophore feature definitions were used in site generation. After the sites were generated hypotheses were generated by a systematic variation of the number of sites. The number of matching active compounds was kept default, that is, entire training set. The process started with five sites, but the set of five-point hypothesis did not survive the scoring process. Gradually the number of sites was reduced to four. Ten hypotheses were generated by the program, out of which only two survived. The scoring was done using the default parameters. The top hypotheses were then used to build pharmacophore-based model. All the molecules were considered active; no thresholds (active or inactive) were applied to the training set for developing the hypothesis. Before considering the model for virtual screening, the hypothesis was overlayed on the enzyme active site and then visually analyzed. After this overlay study, the generated hypothesis was then used to screen the entire DUD dataset. Receptor excluded volume was subsequently considered.

### 2.5. Evaluation Metrics

The virtual screening protocols were validated by their enrichment factors [18–21] and by Receiver operating curve (ROC) [22, 23] analysis. Enrichment factor expresses the number of active compounds found by employing a certain virtual screening strategy. It is a widely used validation tool for assessing the quality of virtual screening protocol. Conceptually the enrichment factor metric is simply the measure of how many more actives we find within a defined “early recognition” fraction of the ordered list relative to a random distribution. The enrichment factors (1) were calculated as follows:

(1) $$\begin{array}{c} E_{f}=\frac{N_{\text{experimental}}x\%}{N_{\text{expected}}x\%}=\frac{N_{\text{experimental}}x\%}{N_{\text{active}}\cdot x\%} \end{array}$$

(*N*_experimental_ is the number of experimentally found active structures in the top *x*% of the sorted database, *N*_expected_ is the number of expected active structures, *N*_active_ is the total number of active structures in database).

ROC curve analysis is considered as one of the best approaches for the performance characterization of virtual screening protocols so far. The ROC is represented equivalently by plotting the fraction of true positives (TPR: true positive rate) versus the fraction of false positives (FPR: false positive rate). 

## 3. Results and Discussion 

### 3.1. Active Site of AChE

**Figure 1:**
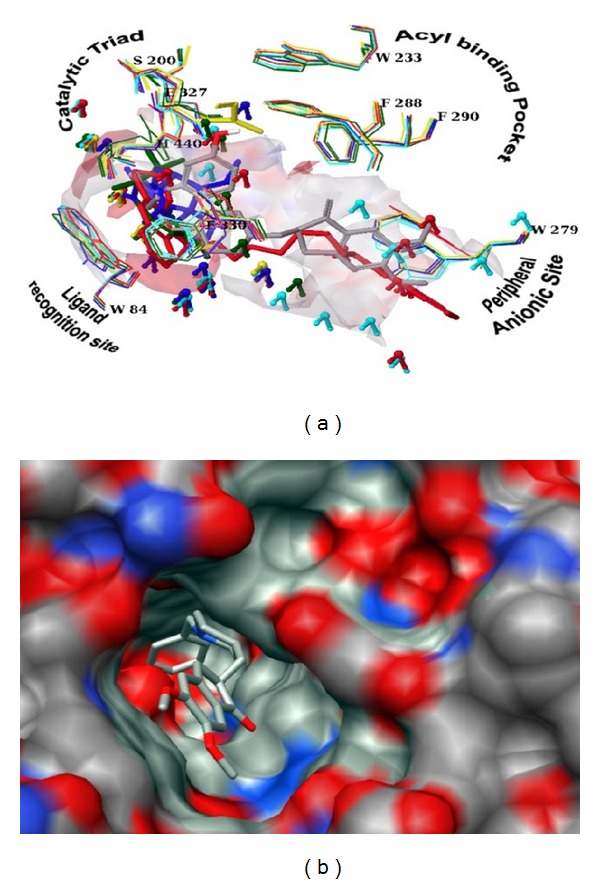
Active site of AChE. (a) Surface view of the active site gorge and donepezil in the active site of PDB ID 1eve. Surface view generated using AutoDock 4.0. (b) Active site of the different PDBs was aligned. Depicting the amino acid residues of different subsites of the active site. Different ligand engages different water molecules. PDBs: 1eve (donepezil): grey; 1gpk (huperzine): blue; 1odc (tacrine): red; 1gqr (rivastigmine): green; and 1ax9 (edrophonium): yellow. Representation: amino acid residues in wire, ligands in tube, and water molecules in ball and stick.

The active site of the enzyme is primarily hydrophobic, subdivided into several subsites: esteratic subsite also called the catalytic triad, acyl binding pocket, peripheral anionic subsite, and ligand recognition site (Figure 1(a)). The active site is buried at the bottom of a 20 Å deep gorge approximately in the centre of the molecule (Figure 1(b)). Different type of ligands binds with different subsites; for example, rivastigmine binds with catalytic triad [24]; donepezil interacts with ligand recognition and peripheral anionic site [25]; huperzine interacts with catalytic triad and ligand recognition site [26]; tacrine interacts with ligand recognition site and peripheral anionic site [27] (Figure 1(a)). Every ligand engages different water molecule(s) [24–27] for binding with the enzyme (Figure 1(a)). 

Thus, it is not practically feasible to choose intrinsic water molecule(s) for structure based virtual screening studies. Thus, the complexity associated with active site of AChE needs a valid model and working algorithms to overcome the virtual screening difficulty. Therefore, exploration of significant search protocol that reliably identifies the subsite where a ligand is most likely to bind is warranted.

When screening a large dataset, a reasonable model encompassing the entire chemical space is essential. If a model of this category is developed, it can easily serve as query tool for screening any dataset. The virtual screening problem for AChE inhibitors was addressed. We have compared different VS tools and discussed the advantages and disadvantages of them. The molecular docking approach was considered for pose analysis and scoring; in addition ligand based strategies were employed for pharmacophore mapping and similarity search. 

### 3.2. Structure Based Studies

**Figure 2:**
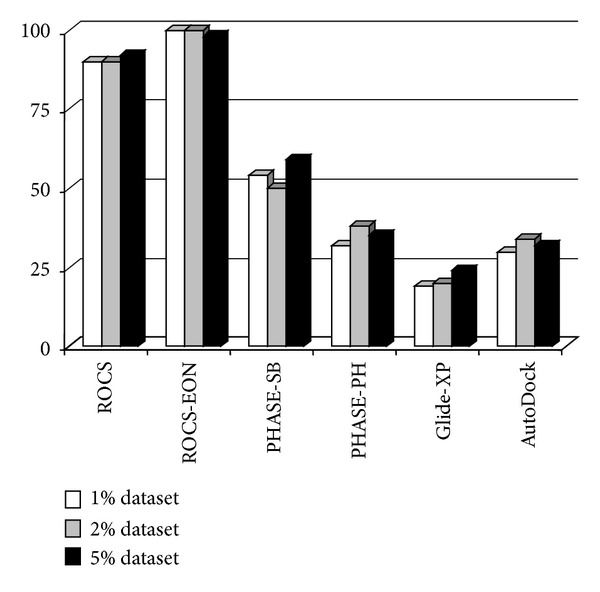
Enrichment factors (EF) calculated at different stages (percent of database screened) of virtual screening. Shape based screening methods such as ROCS and EON outperformed all the other protocols employed.

**Figure 3:**
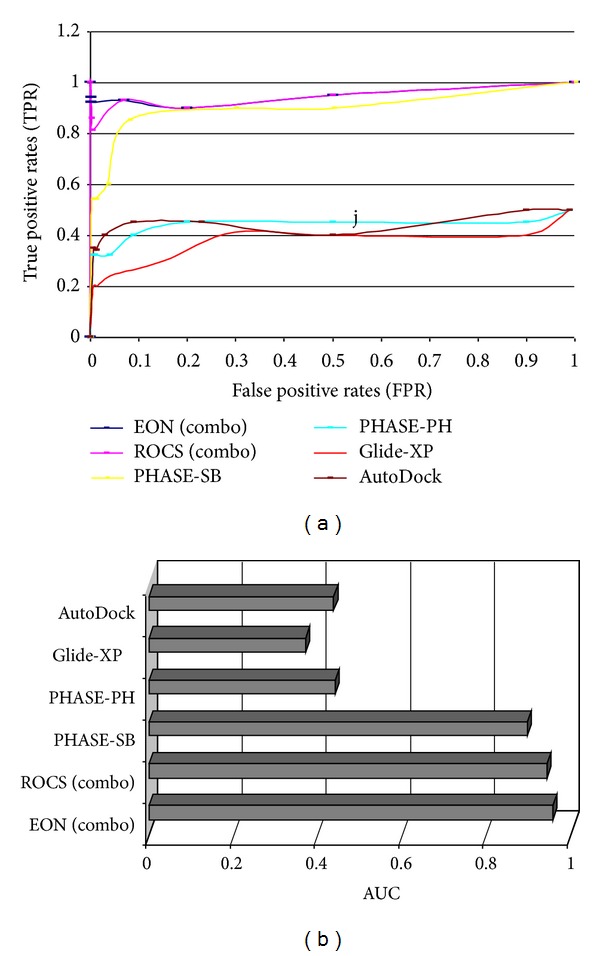
(a) ROC curves for the various protocols employed in the current study. (b) Area under the curve (AUC) values of different protocols used for the virtual screening of AChE. These analyses clearly accentuate the excellent performance of ROCS and EON.

The active site of AChE has a large volume and is divided into many subsites. Therefore, we have used a larger grid (110 × 110 × 110 grid units) that encompasses the entire active site (for both protocols AutoDock and Glide). The results of structure based virtual screening are as follows: for Glide-XP enrichment of 19%, 20% and 24% was observed for top 1%, 2%, and 5% of the dataset, respectively. Whereas, for AutoDock the enrichment was 30%, 34%, and 32%, respectively, for top 1%, 2%, and 5% of the dataset (Figures 2 and 3). 

During our redocking experiment, we observed that increasing the grid size consistently increases the RMSD for both AutoDock and Glide docking. 

**Figure 4:**
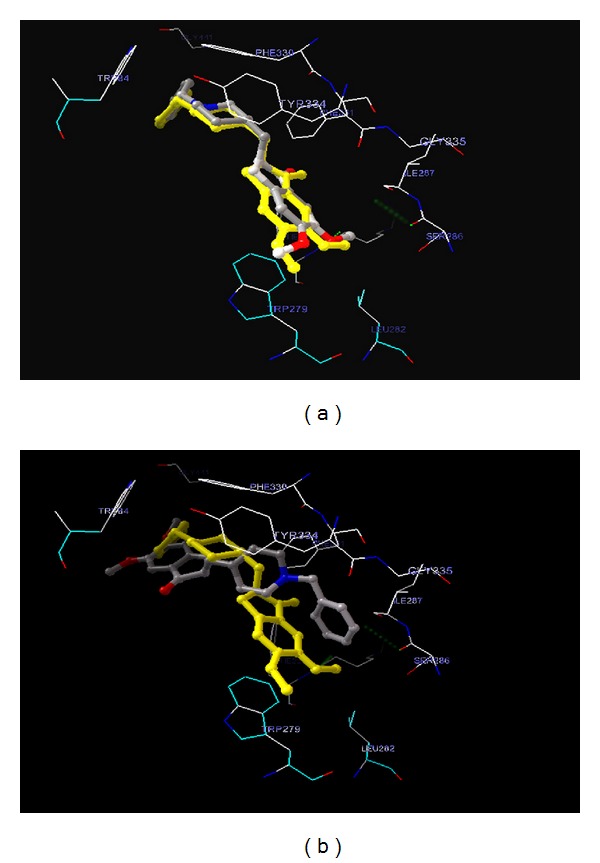
Redocking experiment of donepezil using pdb 1b41. (a) When redocked with a 60 × 60 × 60 grid size, RMSD = 0.69 Å. (b) When redocked with 110 × 110 × 110 grid, RMSD = 4.34 Å.

Taking the example of donepezil, when we redocked with a 60 × 60 × 60 grid size, it yielded an outstanding RMSD of 0.69 Å. Whereas when the same molecule is redocked with 110 × 110 × 110 grid size, it yielded a very poor RMSD of 4.34 Å. The redocked poses are shown in Figure 4. Larger grid presents larger space and more conformational freedom. This increase in space increases the number of interacting pharmacophores, thereby increasing the chance of false interactions. Consequently, this promotes the low enrichment score through molecular docking algorithms.

#### 3.2.1. Effect of Point Mutation on Structure Based Studies

**Figure 5:**
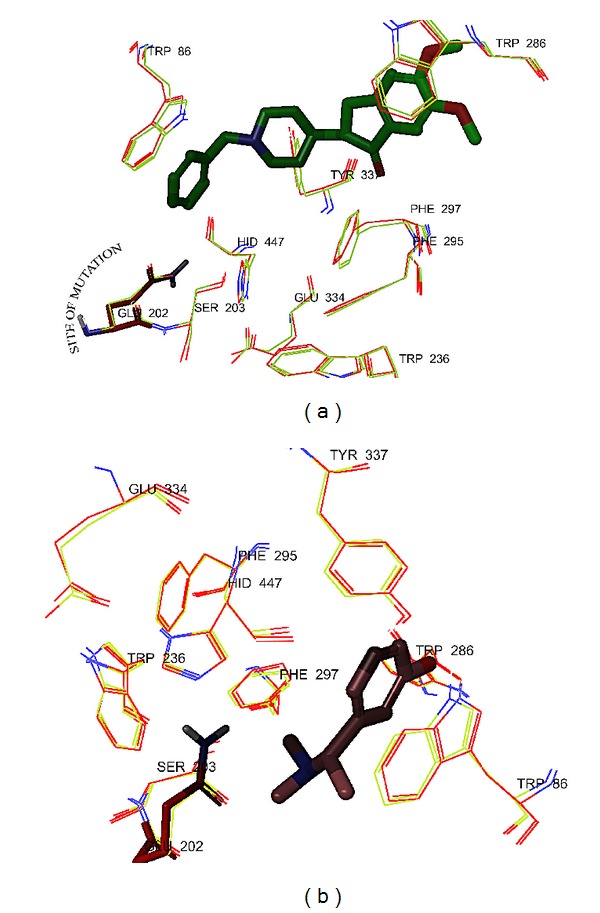
Aligned structures of the wild type human AChE and E202Q mutant AChE. (a) With donepezil (b) With rivastigmine.

We have also studied the effect of docking studies on known mutant of human AChE E202Q. We have used the known ligands and cross-docked them with the mutant form of AChE. As discussed by Krygar and coworkers [25], mutation with neutral GLN in place of charged GLU residue does not change the functional architecture of the active site of AChE. The hydrogen bonding network of GLU202, GLU450, TYR133 is conserved, as a result the architecture and shape of the active site are also maintained. The docking results also correspond to this observation, and no significant changes in docking scores were observed, except rivastigmine. We present a comparison, taking donepezil and rivastigmine as an example (Figure 5).

GLU202 is near to catalytic triad; the presence of this anionic residue is important in molecular recognition for rivastigmine or ligands specific for binding to this triad. Rivastigmine with the protonated nitrogen is naturally attracted towards the anionic site. However, due to mutation in GLU202, we observed a significantly lower affinity for rivastigmine. The affinity of other ligands was unaffected. However, separate studies considering the protein flexibility and intrinsic water molecule(s) are warranted. 

### 3.3. Pharmacophore Modeling

**Figure 6:**
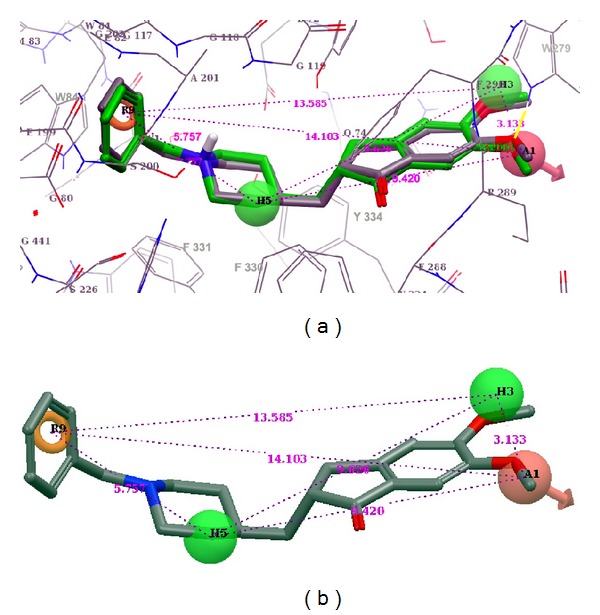
Chosen pharmacophoric hypothesis. (a) Overlaid on the binding site of donepezil bound to PDB ID 1eve. (b) The pharmacophore model depicting the inter-pharmacophoric distances. Ligand structure aligned on the pharmacophore model is donepezil. Hydrogen bond acceptor (A), hydrophobe (H), and aromatic ring (R).

The pharmacophore model was created with the same queries that were used for shape based screening. The model was obtained through an automated mode in PHASE. Before choosing the final model, it was visually inspected through superimposition study on the enzyme active site along with ligand pharmacophore map. Top scoring, four-point AHHR hypothesis was considered further. This generated hypothesis was overlaid on the enzyme active site. The pharmacophoric features were compared with crystallographic information; these interactions were compared and substantiated (Figure 6). 

 A1 (H-bond acceptor): the ligands should have an acceptor group that accepts a hydrogen bond from protonated W279 and H3 and H5 (Hydrophobes); H3 seems to be accommodated in the hydrophobic pocket created by the side chains of W279 and Q74; H5 occupies the hydrophobic pocket created by the four aromatic amino acids F288, 330, 331 and Y334; R9 (ring aromatic feature) is stacked with W84. The interpharmacophoric distances are presented in Table 1. The distance between two extremely located pharmacophores A1 and R9 is 14.103 Å. As described in the earlier section the active site gorge is around 20 Å, around 6 Å deeper than identified by the hypothesis. Therefore, we do not expect to identify those ligands that interact deeper in the active site, during the virtual screening experiment with larger dataset. As expected the enrichment was poor with 32%, 35%, and 38% for the top 1%, 2%, and 5% of the dataset, respectively. The inclusion of excluded volumes did not improve the results (Figures 2 and 3).

### 3.4. Similarity Search

Three computational strategies were employed: PHASE shape-based similarity, ROCS shape based similarity search, and EON electrostatic search. An outstanding enrichment factor (EF) of >90% for analysis by a shape based screening using ROCS was observed in all the cases (1%, 2%, and 5%). However, rescoring of this dataset by EON (ET_combo) improved the results to the best possible outcome. It outperformed all other protocols with outstanding enrichment of >95% in top 1% and 2% of the dataset, with an AUC of 0.958. ROCS (AUC = 0.944) and PHASE-Shape based (AUC = 0.898) protocol performed well, but not as well as EON (ET_combo) (Figures 1 and 2). These results revealed the importance of considering both the electrostatic and shape parameters during the similarity search analysis of AChE inhibitors. 

**Figure 7:**
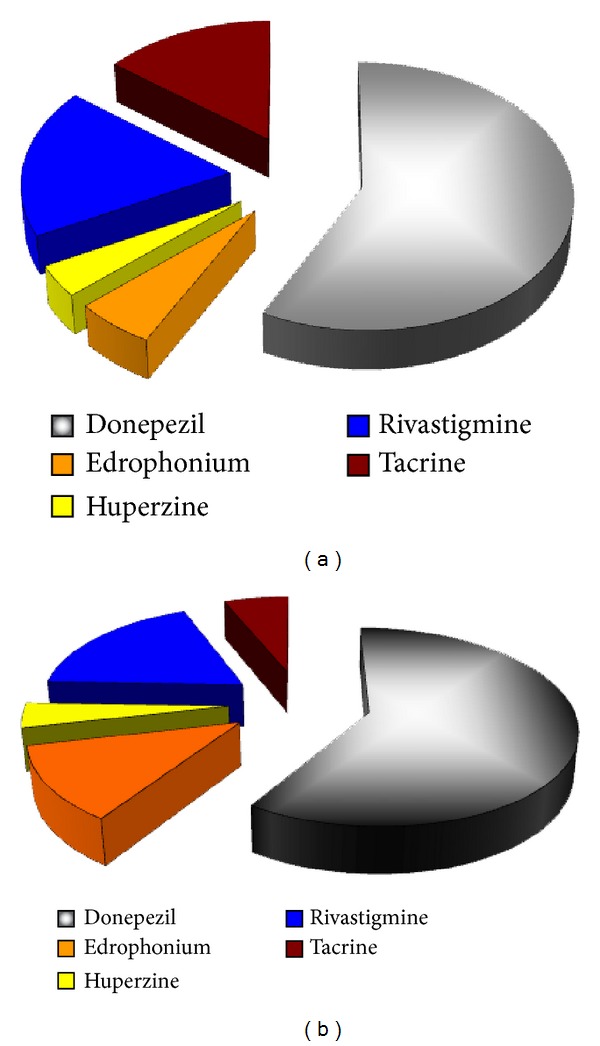
Percentage of different ligands in the top 1% of the dataset during the virtual screening process. (a) Using ROCS. (b) Using EON. It clearly shows the importance of using multiple queries. Using a single query during virtual screening may omit the chemical space encompassed by the others.

The shape based screening protocol adopted in the current study also shows the importance of using multiple queries (Figure 7). Five query ligands were carefully chosen each with different binding mode and occupying different subsite; necessary chemical space is thereby encompassed. Two top models, that is, similarity search using ROCS and ROCS-EON, are scrutinized for influence of individual query molecule on overall quality of the model. Amongst the five queries used (edrophonium, donepezil, huperzine, rivastigmine, and tacrine) in the study, donepezil alone identified 58% and 60% of the entire actives from ROCS and ROCS-EON analysis, respectively. Other query molecules identified around 40% of the actives. If any query molecule is used alone, we would have missed those molecules that are otherwise identified by a different query. Using multiple queries encompasses a much larger chemical space, which is not possible when a single query is employed. 

### 3.5. Comparison with BuChE

We have used five known ligands donepezil, edrophonium, huperzine, rivastigmine, and tacrine. Selectivity towards AChE and BuChE is presented in Table 2. Donepezil [28], huperzine, and edrophonium [29, 30] are selective AChE inhibitors; rivastigmine [31, 32] and tacrine [33, 34] are nonselective. 

Donepezil identified around 60% of the HITs through similarity search. It is a selective AChE inhibitor, so the identified HITs are more likely to be AChE selective inhibitors. On the other hand, tacrine and rivastigmine together identified around 20% of the HITs. These identified HITs are more likely to be nonselective. However, there is no way to prove this hypothesis until we perform biological screening. 

## 4. Conclusions

After an exhaustive validation of various virtual screening methodologies, we have found that shape based search using multiple queries performed better as compared to standard docking studies. The study provided a plausible solution to the virtual screening problem on AChE. We found shape based similarity search methods (ROCS and EON) performed significantly better than structure based methods. The study also revealed the importance of using multiple queries for exploring a larger chemical space and encompassing the majority of the pharmacophoric features. 

## Figures and Tables

**Table 1:** Inter-pharmacophoric distances for the newly developed pharmacophore model.

Site 1	Site 2	Distance
A1	H3	3.133
A1	H5	9.420
A1	R9	14.103
H3	H5	9.626
H3	R9	13.585
H5	R9	5.757

**Table 2:** Comparison of AChE and BuChE selectivity of the selected ligands for similarity search analysis.

S. No.	Compound	AChE *K*_*i*_ (nM)	BuChE *K*_*i*_ (nM)
(1)	Donepezil	2.9	640
(2)	Huperzine	0.026	120
(3)	Tacrine	7	6.9
(4)	Edrophonium	1.6	340000
(5)	Rivastigmine	37	37
